# *Larrea tridentata* Extract Mitigates Oxidative Stress-Induced Cytotoxicity in Human Neuroblastoma SH-SY5Y Cells

**DOI:** 10.3390/antiox8100427

**Published:** 2019-09-25

**Authors:** Karla Morán-Santibañez, Abimael H. Vasquez, Armando Varela-Ramirez, Veronica Henderson, Janae Sweeney, Valerie Odero-Marah, Karine Fenelon, Rachid Skouta

**Affiliations:** 1 Department of Chemistry and Biochemistry, Border Biomedical Research Center, The University of Texas at El Paso, El Paso, TX 79968, USA; ksmoransant@utep.edu (K.M.-S.); ahvasquez@miners.utep.edu (A.H.V.); 2 Border Biomedical Research Center (BBRC), Department of Biological Sciences, University of Texas at El Paso, El Paso, TX 79968, USA; avarela2@utep.edu; 3 Department of Biological Sciences, Center for Cancer Research and Therapeutic Development, Clark Atlanta University, Atlanta, GA 30314, USA; vhenderson@cau.edu (V.H.); janae.sweeney@students.cau.edu (J.S.); voderomarah@cau.edu (V.O.-M.); 4 Department of Biology, University of Massachusetts, Amherst, MA 01003, USA

**Keywords:** oxidative stress, reactive oxygen species, antioxidants, apoptosis, cell cycle, cytoprotection, neurodegenerative diseases, Alzheimer, human neuroblastoma SHSY5Y cells

## Abstract

Creosote bush (*Larrea tridentata*; LT) leaves extracts were tested for their potential efficacy to mitigate cellular oxidative stress on human SH-SY5Y cells. Here, the differential nuclear staining assay, a bioimager system, and flow cytometric protocols, concurrently with several specific chemicals, were used to measure the percentage of cell viability and several facets implicated in the cytoprotective mechanism of LT extracts. Initially, three LT extracts, prepared with different solvents, ethanol, ethanol:water (e/w), and water, were tested for their capacity to rescue the viability of cells undergoing aggressive H_2_O_2_-induced oxidative stress. Results indicate that the LT extract prepared with a mixture of ethanol:water (LT-e/w; 60:40% *v*/*v*) displayed the most effective cytoprotection rescue activity. Interestingly, by investigating the LT-e/w mechanism of action, it was found that LT-e/w extract decreases the levels of H_2_O_2_-provoked reactive oxidative species (ROS) accumulation, mitochondrial depolarization, phosphatidylserine externalization, caspase-3/7 activation, and poly (ADP-ribose) polymerase (PARP) cleavage significantly, which are hallmarks of apoptosis. Thus, out of the three LT extracts tested, our findings highlight that the LT-e/w extract was the most effective protective reagent on SH-SY5Y cells undergoing oxidative stress in vitro, functioning as a natural anti-apoptotic extract. These findings warrant further LT-e/w extract examination in a holistic context.

## 1. Introduction

Neurodegenerative diseases (NDs) are characterized by progressive deterioration of the structure and function of the central nervous system that leads to compromise motor or cognitive function [1,2]. Oxidative stress due to the excessive production of reactive species, especially reactive oxygen (ROS) and nitrogen (RNS) species besides the failure of balance by antioxidant enzyme systems, have been involved in the development of Alzheimer’s disease (AD), Parkinson’s disease (PD), and other NDs [3,4]. 

Oxidative damage in the cell manages the destruction of biomolecules such as lipids, proteins, and DNA, so counteracting the effects produced by this oxidative stress is one of the approaches to neuroprotection [5]. Neuroprotection can be defined as the relative strategies and mechanisms that defend the central nervous system for neuronal injuries due to both acute (e.g., stroke or trauma) and chronic neurodegenerative diseases [6]. 

The lack of successful therapies to overcome oxidative stress without side effects has prompted the research of new treatments. Recently, the main approaches of new therapies have focused on three main sources: Synthesis of new drugs, drug repurposing, and natural products research [7,8]. From natural products, plant extracts have been specially studied and proven to be effective for memory enhancement, anti-aging, and neuroprotective purposes [9,10,11]. 

Polyphenolic compounds are secondary metabolites widely distributed in plants and have been considered of interest for their potential benefit to human health [12]. Many biological activities have been attributed to phenolic compounds, among which are an antitumor, immunomodulatory, antiviral, antibacterial, and antioxidant activity [13,14,15,16]. Previous research has shown that polyphenolic compounds directly eliminate ROS and offer antioxidant effects that are beneficial for several diseases, including NDs [17].

In a previously published study, we evaluated the antioxidant activity of extracts of *Larrea tridentata*, a plant commonly used in traditional medicine for its antiviral, antiparasitic, anti-inflammatory, and antioxidant properties [18,19,20]. Among the three evaluated extracts, we found that the ethanol:water extract had the most efficient antioxidant properties and the highest phenolic content. The high-performance liquid chromatography-mass spectrometry (HPLC-MS) study of this extract identified phenolic compounds previously reported in *Larrea tridentata* [18]. 

In the present study, we tested the cytoprotective activity of an extract *of Larrea tridentata* (ethanol:water) in an in vitro model of H_2_O_2_-induced oxidative stress and apoptosis in human SH-SY5Y cells.

## 2. Materials and Methods

### 2.1. Chemicals and Reagents

Dulbecco’s Modified Eagle Medium (DMEM)/F-12 (Gibco Invitrogen, Carlsbad, CA, USA), fetal bovine serum (FBS; Gibco Invitrogen, Carlsbad, CA, USA), and Penicillin-Streptomycin (Lonza, Walkersville, MD, USA) were used for cell culture assays. Hoechst 33342 (Hoechst; blue signal; Invitrogen, Eugene, OR, USA) and propidium iodide (PI; red signal; MP Biomedicals, Solon, OH, USA) were used for cytotoxicity assays. MitoProbe™ JC-1 Assay Kit (ThermoFisher Scientific Inc., Rockford, IL, USA) was used for measuring the mitochondrial membrane potential. Carboxy-H2DCFDA (ThermoFisher Scientific Inc., Rockford, IL, USA) was used to detect intracellular reactive oxygen species. Dead Cell Apoptosis Kit with Annexin V FITC and PI (ThermoFisher Scientific Inc., Rockford, IL, USA) was used to detect apoptosis. NucView 488 Caspase-3 kit for live cells (Biotium, Hayward, CA, USA) was used to detect Caspase-3 activity. For Western Blot analysis, mouse monoclonal anti-human actin antibody (Sigma-Aldrich Inc., St. Louis, MO, USA) and Rabbit monoclonal anti-cleaved poly (ADP-ribose) polymerase (PARP; Cell Signaling Technology, Inc., Danvers, MA, USA) were used. Horseradish peroxidase (HRP)-conjugated sheep anti-mouse, donkey anti-rabbit secondary antibodies, and the Enhanced Chemiluminescence (ECL) western blotting detection reagent were from GE Healthcare UK Ltd. (Buckinghamshire, UK). Nitrocellulose membranes were from Bio-Rad Life Sciences Research (Hercules, CA, USA). Total protein content was determined using the BCA protein assay kit (Thermo Scientific, Rockford, IL, USA). Cell cycle analysis was performed using a nuclear isolations and staining solution (NIM-DAPI 731085) (NPE systems, Pembroke Pines, FL, USA).

### 2.2. Preparation of Larrea tridentata (LT) Extracts

Leaves from creosote bush, *Larrea tridentata* (LT) were field collected from the Chihuahuan desert in the region of El Paso del Norte, TX, USA dried, and triturated to a fine powder. Authentication of collected samples was assessed by Professor Emeritus Richard D. Worthington, a plant biodiversity expert at the University of Texas at El Paso. The LT extracts were essentially prepared as previously detailed [18]. Briefly, dehydrated powder of LT leaves was resuspended with ethanol:water (60:40% *v*/*v*) mixture and kept at 40 °C for 4 h, followed by incubation at room temperature for 24 h. Then, LT leaves in ethanol:water (e/w) suspensions were filtered using Celite 545 (Millipore Sigma, St. Louis, MO, USA) to remove the leaf material and the filtered solution was collected as a dark brown oil and stored at 4 °C for further experimentation. Additionally, two LT extracts were prepared using just ethanol or just water as described above. Furthermore, the LT ethanol:water extract was now denoted as LT-e/w extract. The consistency of the original LT-e/w extract was highly viscous, and to increase its solubility, it was necessary to add dimethyl sulfoxide (DMSO) to prepare stock solutions easy to manipulate via pipetting instruments. Thus, DMSO at the final concentration 0.25% *v*/*v* was consistently used as a solvent control in all the experiments included in this study, because that was the final concentration contained in the LT-e/w extract experimental samples. The fractionation of the whole LT-e/w extract was conducted using a HPLC system. The parameters applied during this fractionation strategy were as described previously [18]. Our previous reported study of the fractionation of the LT-e/w extract using HPLC allowed the separation of nine fractions [14]. The mass spectroscopy (MS) analysis, supported by the available online Mass Bank, a public repository for sharing mass spectral data for life sciences, allowed the tentative identification of different compounds (see Table 3 in Ref. [14]). In general, most of the identified compounds (e.g., tuglanin, tyramine, justicidin B, eleutherol, 3′,4′,5,7-tetraacetoxyflavone, 3′,4′,5,7-tetramethylquercetin, liquiritin, podophyllotoxin, and beta peltain) are natural phenolic compounds with antioxidant activity that have been previously reported in other plants related to *Larrea tridentata* [14]. Moreover, justicidin B and beta peltain were among the most abundant identified compounds, which have previously been listed as secondary metabolites of *Larrea tridentata*.

### 2.3. Cell Culture

Human neruroblastoma SH-SY5Y cells (ATCC^®^ CRL-2266^™^) were purchased from ATCC (Manassas, VA, USA). Cells were grown using Dulbecco’s Modified Eagle Medium (DMEM)/F-12 nutrient mixture supplemented with 10% heat-inactivated fetal bovine serum (FBS; Corning, Corning, NY, USA), 100 U/mL penicillin, and 100 μg/mL streptomycin (Lonza, Walkersville, MD, USA). Consistently, the incubation conditions used to grow were 37 °C in a humidified 5% CO_2_ atmosphere.

### 2.4. Cytotoxicity Assays

In order to evaluate the cytotoxicity effect of LT extracts, SH-SY5Y cells were seeded in 96-well plates at a density of 1 × 10^4^ cells/well in 100 µL of growing media and incubated overnight before starting the treatments. Next, increasing concentrations of LT extracts (0.25% DMSO highest final concentration) were added to the cells. The differential nuclear staining (DNS) assay, using Hoechst 33342 and propidium iodide (PI) dyes (5 µg/mL final concentration each), was performed to evaluate cytotoxic activity [21,22,23]. Likewise, the ability of LT extracts to protect SH-SY5Y cells from H_2_O_2_-induced apoptosis was determined. SH-SY5Y cells were treated with H_2_O_2_ over the concentration range of 75–600 μM up to 12, 18, and 24 h to standardize H_2_O_2_ treatment used for the determination of cytoprotective effects. Once the best conditions were determined for the cytoprotection assay, cells were treated concurrently with increasing concentrations of LT extracts (7.5–30 μg/mL) and 600 µM H_2_O_2_ for 12 h. After that incubation period, the cytotoxicity was measured as described previously. Also, after LT-e/w extract was fractionated using an HPLC system, four (1 to 4) of the most prominent fractions were individually reviewed for their cytoprotective activity as described above. Each experimental point and controls were assessed by three independent measurements; 0.25% *v*/*v* DMSO, 600 µM, and 20 mM H_2_O_2_ were used as controls.

### 2.5. Mitochondrial Membrane Potential (ΔΨm) Assay

Polychromatic analysis of mitochondrial membrane potential (*ΔΨm*) was determined using the fluorescent probe, JC-1 (ThermoFisher Scientific). SHSY5Y cells were treated for 6 h with LT-e/w extract (7.5 and 15 µg/mL) and 600 µM H_2_O_2_. After this period, cells were harvested using 0.1% trypsin-EDTA and cell pellets were treated according to the manufacturer’s instructions. As a control, 0.25% DMSO, 600 µM H_2_O_2_, were used. The green fluorescence signal emission of JC-1 monomeric form is indicative of mitochondrial depolarization. The samples were analyzed using Gallios Flow Cytometer (Beckman Coulter. Miami, FL, USA) assisted with Kaluza software. Around 10,000 cells (events) were acquired and analyzed per sample. Each experimental point and controls were assessed by three independent measurements.

### 2.6. ROS Production Assay

The SHSY5Y cells were treated for 15 min with carboxy-H_2_DCFDA (2 µg/mL) following a cotreatment of LT-e/w extract (7.5 and 15 µg/mL) and 600 µM H_2_O_2_. After periods of 1 h and 4 h of incubation, cells were harvested using 0.1% trypsin-EDTA. Intracellular ROS production was evaluated based on the oxidation of carboxy-H_2_DCFDA detected by monitoring the increase in fluorescence with a flow cytometer Gallios Flow Cytometer (Beckman Coulter) assisted with Kaluza software. The samples were analyzed using 0.25% DMSO, 600 µM, and 20 mM H_2_O_2_ as controls. Each experimental point and controls were assessed in triplicate.

### 2.7. Annexin V-FITC and Propidium Iodide Staining Assay

To determine the protective effect of LT extracts on early/late apoptosis and necrosis induced by H_2_O_2_, SH-SY5Y cells were stained with fluorescein isothiocyanate (FITC)-conjugated Annexin V and propidium iodide. SH-SY5Y cells were cultured in 24-well plates at a density of 1 × 10^5^ cells/well at 37 °C in a 5% CO_2_ atmosphere. After overnight incubation, cells were treated with LT-e/w extract (7.5 and 15 µg/mL) and 600 µM H_2_O_2_ and incubated for an additional 12 h. Then, cells were harvested using 0.1% trypsin-EDTA and cell pellets were resuspended with 100 µL of ice-cold 1× binding buffer containing annexin V-FITC solution and propidium iodide following the manufacturer’s instructions (Thermo Fisher Scientific). The cell samples were analyzed using Gallios Flow Cytometer (Beckman Coulter) assisted with Kaluza software [24]. Each experimental point and controls were assessed in triplicate, and 0.25% DMSO, 600 µM, and 20 mM H_2_O_2_ were used as controls.

### 2.8. Caspase-3 Activity Analysis

Live-cell detection of intracellular caspase-3 activation in SH-SY5Y treated with LT (ethanol:water extract) and H_2_O_2_ was determined using a fluorogenic NucView 488 Caspase-3 kit for live cells (Biotium, Hayward, CA, USA). SH-SY5Y cells were seeded on a 24-well plate format as described above and were treated for 8 h with LT-e/w extract (7.5 and 15 µg/mL) and H_2_O_2_ 600 µM; 0.25% DMSO, 600 µM and 20 mM H_2_O_2_ were used as controls. Cells were harvested using 0.1% trypsin-EDTA and cell pellets were treated according to the manufacturer’s instructions. The samples were analyzed using Gallios Flow Cytometer (Beckman Coulter), data collection, and analysis of the caspase-3-positive cells was performed using Kaluza software. Each experimental point and controls were assessed by three independent measurements.

### 2.9. Western Blot Analysis

Mouse monoclonal anti-human actin antibody was from Sigma-Aldrich Inc. (St. Louis, MO, USA). Rabbit monoclonal anti-cleaved PARP was from Cell Signaling Technology, Inc. (Danvers, MA, USA). HRP-conjugated sheep anti-mouse, donkey anti-rabbit secondary antibodies, and the Enhanced Chemiluminescence (ECL) western blotting detection reagent were from GE Healthcare UK Ltd. (Buckinghamshire, UK). Nitrocellulose membranes were from Bio-Rad Life Sciences Research (Hercules, CA, USA). Western blot was performed as described previously [25]. Cells were lysed with buffer containing 1 × RIPA, with protease inhibitors (aprotinin 0.1 mg/mL, PMSF 1 mM, leupeptin 0.1 mM, pepstatin A 0.1 mM) and phosphatase- inhibitor (10 mM sodium orthovanadate) and centrifuged at 13,000 rpm. Total protein content was determined using the BCA protein assay kit (Thermo Scientific, Rockford, IL, USA) following protocol as per the manufacturer’s specifications. Cell lysates (40–50 µg) were subjected to SDS-Polyacrylamide gel electrophoresis (SDS-PAGE) and subsequently transferred to pure nitrocellulose membrane. Western blot analysis for cleaved-PARP was then performed. Anti-rabbit IgG horseradish peroxidase in blocking solution (5% nonfat milk in TBS-Tween 20) was employed as a secondary antibody for chemiluminescent detection. Blots were visualized with chemiluminescence ECL detection system (Pierce, Rockford, IL, USA) and analyzed using FUJIFILM LAS 3000 imager and darkroom X-ray film to observe protein expression. To evaluate protein loading, membranes were immediately stripped with Restore TM Western Blot Stripping Buffer (Thermo Scientific) and reprobed for β-actin as a loading control.

### 2.10. Cell Cycle Analysis

Cell cycle progression of SH-SY5Y cells treated with LT-e/w extract and/or H_2_O_2_ was determined by flow cytometric measurement of total cellular DNA content. SH-SY5Y were seeded on a 24-well plate format as described above and treated for 12 h with LT-e/w extract (7.5 and 15 mg/mL), and 600 µM H_2_O_2_; 0.25% DMSO, 600 µM H_2_O_2_, 100 nM etoposide, and 1 mg/mL G418 were used as controls. Cells were harvested using 0.1% trypsin-ethylenediaminetetraacetic acid (EDTA) and cell pellets were stained with nuclear isolations and staining solution (NIM-DAPI 731085) according to the manufacturer’s protocol (NPE systems). Data acquisition was achieved using a Gallios flow cytometer (Beckman Coulter). Approximately 20,000 cells (events) were acquired and analyzed per sample. The percentage of cell subpopulations in each cell cycle phases was analyzed via Kaluza software (Beckman Coulter). Each experimental point and controls were assessed in triplicate.

### 2.11. Statistical Analysis

All experiments were performed at least in triplicate. Data are shown as average with its corresponding standard deviation to denote experimental variability. To determine the statistical significances between two experimental samples, two-tailed paired Student’s *t*-tests were performed (http://www.socscistatistics.com/tests/studentttest/Default2.aspx). A value of *p* < 0.05 was deemed significant to designate whether comparisons of two samples have statistical significance. 

## 3. Results

### 3.1. LT-e/w Extract Protected SH-SY5Y Cells Against H_2_O_2_-Induced Cytotoxicity

**Figure 1:**
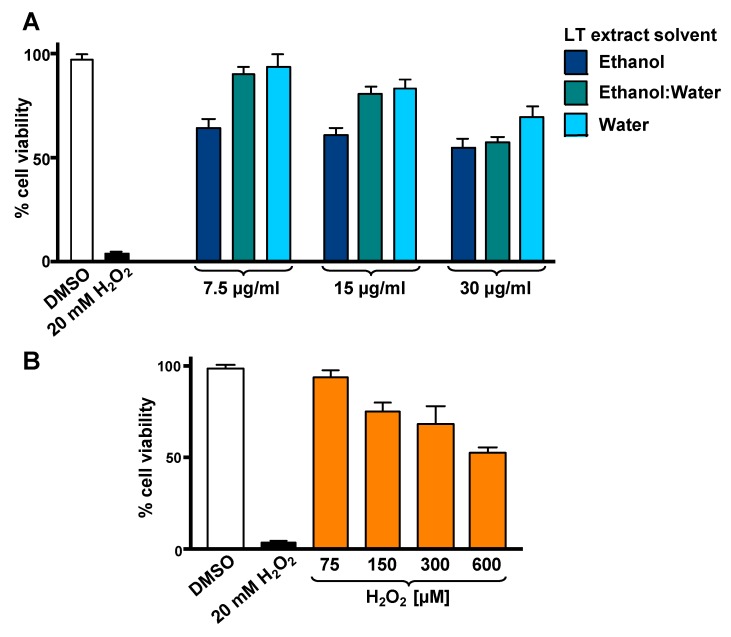
Cytotoxicity of *L. tridentata* (LT) extracts (**A**) and H_2_O_2_ (**B**) on SH-SY5Y cells. The percentage of viable cells was measured using the differential nuclear staining (DNS) assay and a bioimager system. (**A**) Cells were treated for 24 h with a concentration gradient (7.5, 15 and 30 µg/mL) of each LT extract dissolved with ethanol, ethanol:water (e/w), or water. (**B**) The percentage of viable cells exposed for 12 h to an H_2_O_2_ concentration gradient (75 to 600 µM) was also determined. Dimethyl sulfoxide (DMSO) 0.25% *v*/*v* was included as a control. As a positive control for cytotoxicity, 20 mM of H_2_O_2_-treated cells were also included. Each bar indicates the average of three biological replicates with its corresponding standard deviation.

**Figure 2:**
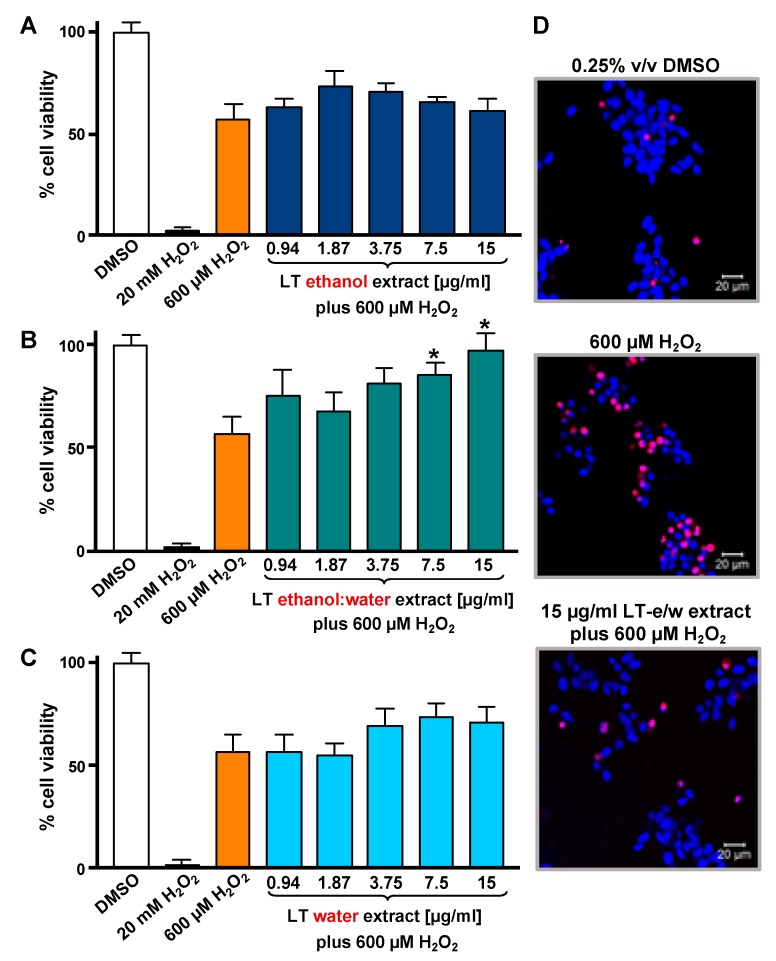
LT extracts prepared with three different solvents were tested on SH-SY5Y cells under H_2_O_2_-induced oxidative stress. The DNS assay and a bioimager system were used. Cells were exposed for 12 h to both a single H_2_O_2_ concentration (600 µM) and a concentration gradient (0.94 to 15 µg/mL) of the LT extracts in different solvents: (**A**) Ethanol, (**B**) ethanol:water (e/w) mixture, and (**C**) water. Controls included cells treated with DMSO (0.25% *v*/*v*) and 20 mM of H_2_O_2_ as positive for cytotoxicity. The asterisk (*) indicates a significant difference between cells treated with both LT-e/w extract and H_2_O_2_ (600 µM), as compared with cells treated just 600 µM H_2_O_2_ control (*p* < 0.05). Each bar shows the average of three biological replicates with its corresponding standard deviation. (**D**) Representative images of SH-SY5Y cells stained with Hoechst (blue) and PI (red) used to quantify levels of cytotoxicity: Vehicle control (DMSO); positive control for cytotoxicity, 600 µM H_2_O_2_; and cotreatment with both LT-e/w extract and 600 µM H_2_O_2_. Cells emitting just blue signal were considered healthy/alive, whereas cells emitting magenta color, as a result of colocalization of the blue and red fluorescence signal, were considered dead cells.

To optimize the incubation time and concentrations utilized in the cytotoxicity rescue experiments, either LT extracts or H_2_O_2_ several were tested independently on SH-SY5Y cells, measuring their viability using DNS assay and a bioimager system. Initially, cells exposed for 24 h to a concentration gradient (7.5 to 30 µg/mL) of the three LT extracts, ethanol, ethanol:water (e/w) mix, and water, were tested (Figure 1A). From these experiments, the concentrations of 7.5 and 15 µg/mL of LT-e/w extracts were selected for further experiments (Figure 1A). Also, the cytotoxic effect of a concentration gradient of H_2_O_2_ was investigated on SH-SY5H cells by incubating for 12 h (Figure 1B). In addition, a concentration gradient of H_2_O_2_ was also tested at 18 h (Figure S1A) and 24 h (Figure S1A), respectively. From these series of experiments, cells treated for 12 h with 600 µM of H_2_O_2_ were selected for subsequent rescue experiments, as the percentage of its cytotoxicity observed was around 50%, as compared with solvent-treated cells (Figure 1B). To determine the cytoprotective activity of the LT extracts, cells were co-exposed to both an LT extract single concentration plus 600 μM H_2_O_2_, and the percentage of cell viability was compared to cells treated with 600 μM of H_2_O_2_ alone after 12 h (Figure 2). Findings indicated that the LT-e/w extract tested at 15 µg/mL was the most effective and exerted the most the cytoprotective activity, as evidenced by a significant increase in cell viability (*p* < 0.01; Figure 2B). Also, a series of experiments using a combination of 150 µM or 300 µM of H_2_O_2_ together with a concentration gradient of each LT extract incubated for 12 h, 18 h, and 24 h were performed (Figures S2–S6). A corollary to these data suggests that the H_2_O_2_ induces cytotoxicity in time and dose-dependent manner and LT-e/w was the most efficient cytoprotective extract, as evidenced by a significant rescue activity when tested at 15 µg/mL for 12 h of incubation. Also, this LT-e/w extract rescue activity on cells under oxidative stress was a time and dose-dependent occurrence. 

### 3.2. Four Fractions From LT-e/w Extract Exhibit Undetected Protective Activity on H_2_O_2_-Induced Toxicity on SH-SY5Y Cells

**Figure 3:**
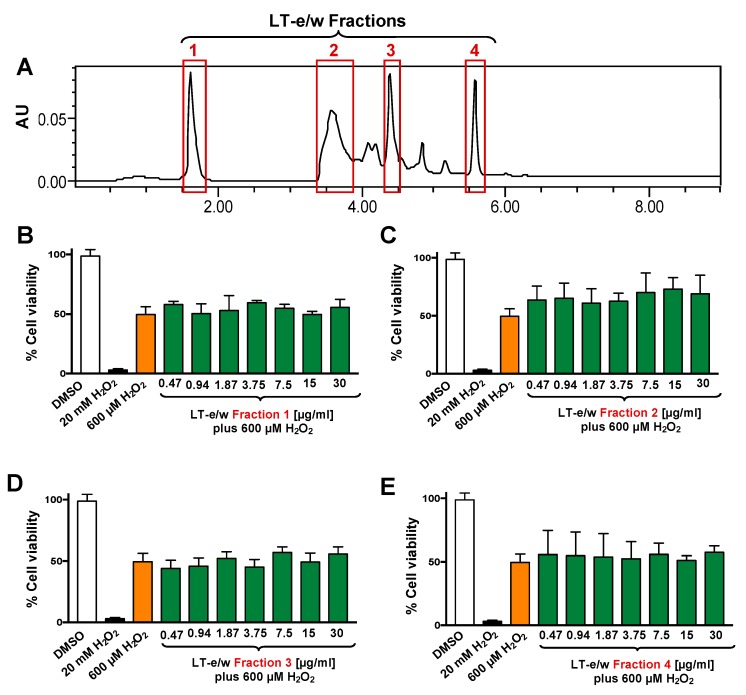
Four LT-e/w fractions were collected and individually tested on SH-SY5Y cells under H_2_O_2_-induced oxidative stress. (**A**) Chromatogram obtained from the whole LT-e/w extract, where the red rectangular boxes specify the choices for all the four fractions. The four most prominent peaks were individually collected and named from 1 to 4. Cells were exposed for 12 h to both a single H_2_O_2_ concentration (600 µM) and a concentration gradient (0.47 to 30 µg/mL) of each individual LT-e/w fraction: (**B**) Fraction 1, (**C**) Fraction 2, (**D**) Fraction 3, and (**E**) Fraction 4. The following controls were included in this series of experiments: Cells treated with DMSO (0.25% *v*/*v*) as solvent control, and cells exposed to 20 mM of H_2_O_2_ as a positive control for cytotoxicity. Each bar shows the average of three biological experiments with its corresponding standard deviation.

The total LT-e/w extract was fractionated via HPLC system (Figure 3). Four (1 to 4) of the most prominent fractions were tested for their antioxidant rescue activity as described above. Findings indicated that none of the four selected LT-e/w fractions exhibited significant cytoprotection activity on SH-SY5Y cells under oxidative stress (Figure 3).

### 3.3. LT-e/w Extract Reduced The H_2_O_2_-Induced Mitochondrial Depolarization 

**Figure 4:**
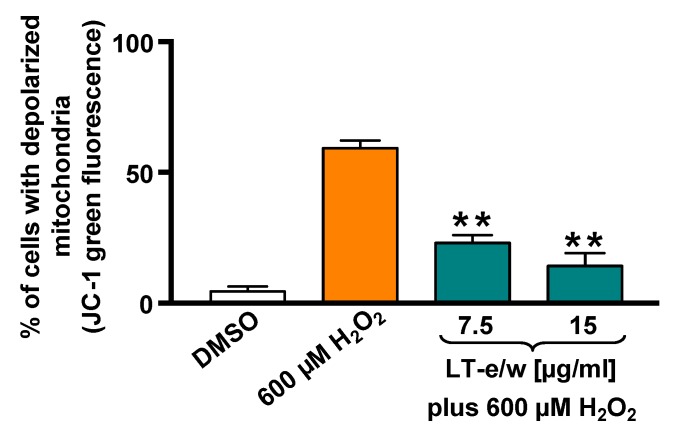
LT-e/w extract decreased the H_2_O_2_-induced mitochondrial depolarization significantly on SH-SY5Y cells in a concentration-dependent mode. Mitochondrial membrane potential (*ΔΨm*) changes were measured by the JC-1 staining profile via flow cytometer. DMSO 0.25% *v*/*v* was included as a control. As a positive control for cytotoxicity, 600 µM of H_2_O_2_-treated cells were also included. The y-axis displays the percentage of cells with depolarized mitochondria emitting the JC-1 green fluorescence signal. The significant difference between 600 µM H_2_O_2_-treated cells, as compared with cells simultaneously treated with both LT-e/w extract plus 600 µM H_2_O_2_, was of *p* < 0.01 (**). Each bar indicates the average of three biological replicates with its corresponding standard deviation.

The SH-SY5Y cells cotreated with LT-e/w extract (7.5 µg/mL and 15 µg/mL) and 600 μM H_2_O_2_ were stained with JC-1 probe to determine mitochondrial membrane potential (*ΔΨm*). The red fluorescent signal due to JC-1 aggregates in membrane depolarization was detected in SH-SY5Y cells. Figure 4 shows that a significant increment in mitochondrial depolarization was determined in cells exposed to 600 µM H_2_O_2_ (*p* < 0.01 compared with DMSO control). LT-e/w extract interferes with the formation of JC-1 aggregates and significantly reduced the H_2_O_2_-induced mitochondrial depolarization in cotreated cells, as compared with just 600 μM H_2_O_2_ control (*p* < 0.01). These results suggest that LT-e/w extract is effectively reducing the mitochondrial depolarization in SH-SY5Y cells, further indicating its anti-apoptosis property.

### 3.4. LT-e/w Extract Attenuates H_2_O_2_-Induced Increase of Intracellular ROS Levels

**Figure 5:**
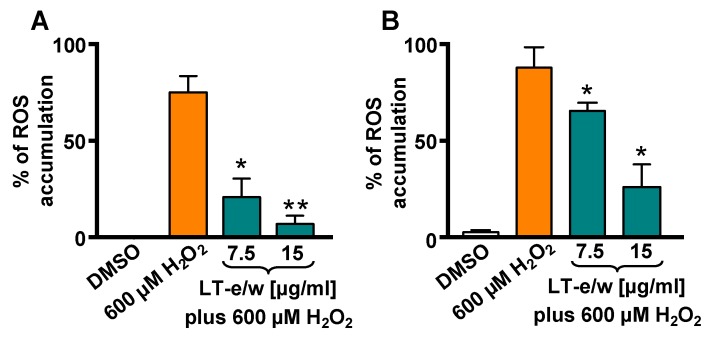
LT-e/w extract diminished the reactive oxygen species (ROS) accumulation significantly in SH-SY5Y cells under H_2_O_2_-induced oxidative stress. Cells were exposed to carboxy-H_2_DCFDA for 15 min, following by 1 h (**A**) and 4 h (**B**) of incubation with diverse treatments. Subsequently, the ROS accumulation was measured via flow cytometer. Cells treated with DMSO or with 600 µM of H_2_O_2_ were included as controls. Each bar shows the mean of three biological replicates with its corresponding standard deviation. The significance of differences between 600 µM H_2_O_2_-treated cells, as compared to cells treated concomitantly with both LT-e/w extract plus 600 µM H_2_O_2_, were of *p* < 0.05 (*) or *p* < 0.01 (**), respectively.

Intracellular ROS levels were measured by the oxidation of carboxy-H_2_DCFDA reagent in SH-SY5Y cells via flow cytometer. A highly significant increment in ROS accumulation was determined in cells exposed to 600 µM H_2_O_2_ (*p* < 0.01), as compared with DMSO control (Figure 5). Cells concurrently exposed for 1 h to both 600 μM of H_2_O_2_ and 7.5 µg/mL or 15 µg/mL of LT-e/w extract showed a significant reduction in ROS, as compared with 600 µM H_2_O_2_-treated cells (control), *p* < 0.05 or *p* < 0.01, respectively (Figure 5A). In addition, cotreated cells for 4 h also showed a reduction of ROS accumulation, as compared with cells treated with just H_2_O_2_ control (*p* < 0.05; Figure 5B). Thus, LT-e/w extract was capable of effectively attenuating ROS accumulation in a concentration- and time-dependent manner, suggesting that LT-e/w extract could be a potent free radical scavenger. 

### 3.5. LT-e/w Extract Interfered With H_2_O_2_-Induced Apoptosis In SH-SY5Y Cells

**Figure 6:**
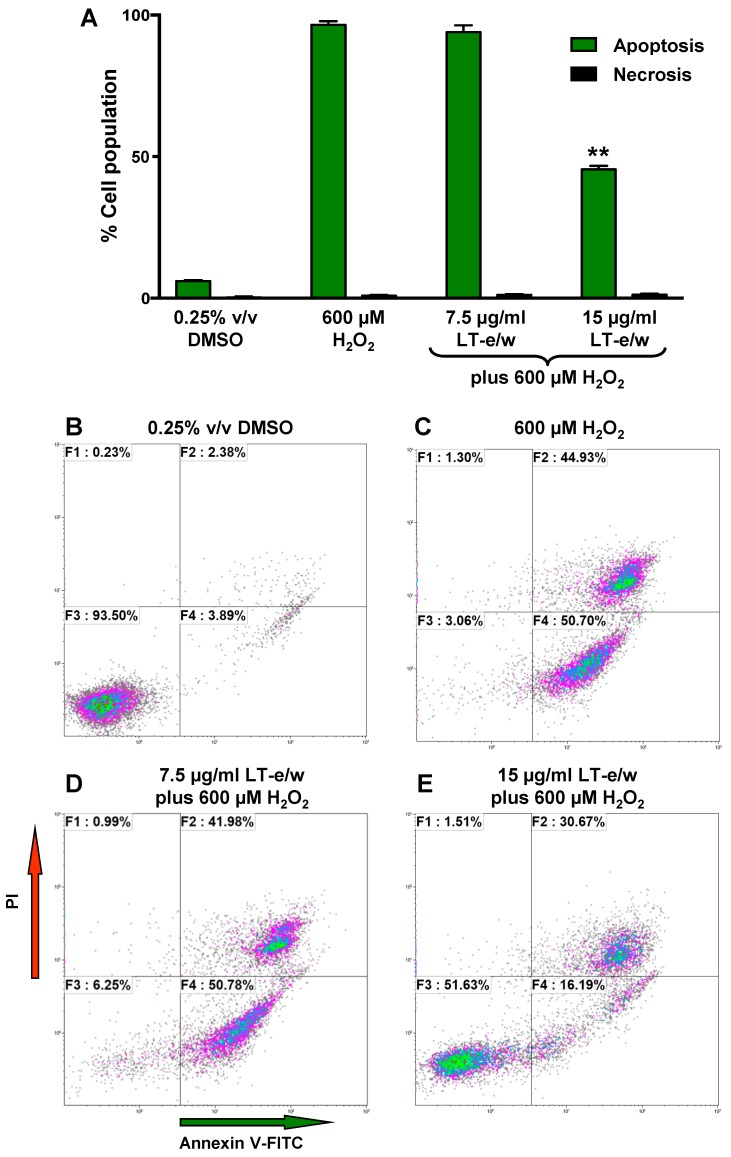
LT-e/w extract reduced the H_2_O_2_-induced apoptosis significantly in SH-SY5Y cells in a dose-dependent manner. (**A**) After the cells were concurrently exposed to LT-e/w extract (7.5 or 15 µg/mL) and 600 μM H_2_O_2_, the annexin V-FITC, and propidium iodide (PI) staining profile was reviewed via flow cytometer (Gallios, Beckman Coulter). Each bar specifies the average of three biological replicates with its corresponding standard deviation. The asterisk (**) indicates a significant difference between cells exposed to both 15 µg/mL LT-e/w extract and 600 µM H_2_O_2_, as compared with cells treated with just 600 µM H_2_O_2_ (*p* < 0.01). B to E) Representative flow cytometric dot plots used to quantify the apoptosis and necrosis percentages depicted in panel A. (**B**–**E**) Analysis from each quadrant in the dot plots were defined as follows: The bottom left quadrant denotes unstained viable cells, annexin V-FITC, and PI negative; the top left quadrant specifies necrotic cells that are just PI-positive; the top right quadrant comprises late apoptotic cells that are both annexin V-FITC- and PI-positive; and the right bottom quadrant indicates early apoptotic cells that are just positive for annexin V-FITC. The Kaluza flow cytometry software (Beckman Coulter) was used for acquisition and analysis of data.

Under homeostasis, phosphatidylserine (PS) is localized in the inner leaflet of the plasma membrane facing the cytosol. However, under apoptosis, the plasma membrane asymmetry is compromised and PS is externalized to the surface of the cells [26]. This biochemical event is considered a hallmark of apoptosis. PS externalization induced by H_2_O_2_ treatments was investigated using annexin V-FITC/propidium iodide (PI) staining assay and monitored flow cytometer. In SH-SY5Y cells, exposure to H_2_O_2_ showed significant differences in an increase of apoptotic cells (98%), as compared to solvent control (5%; Figure 6). Cotreated cells (LT-e/w extract 15 µg/mL and 600 μM H_2_O_2_) exhibited 45% annexin V-FITC positive cells, in contrast with 98% when treated with 600 μM H_2_O_2_ alone (Figure 6). This result indicated that H_2_O_2_ at 600 μM was toxic to SH-SY5Y cells. However, co-exposure with LT-e/w extract showed cytoprotective effects against H_2_O_2_-induced apoptosis. Differences in the numbers of necrotic cells in both experimental and control cells were insignificant. Thus, findings suggest that the LT-e/w extract possess an anti-apoptotic effect in a dose-dependent mode, as evidenced by the reduction of the number of annexin V-FITC positive cells provoked by aggressive oxidative stress. 

### 3.6. The LT-e/w Extract Cytoprotective Effect Involves a Decrease of Caspase-3 Activation

**Figure 7:**
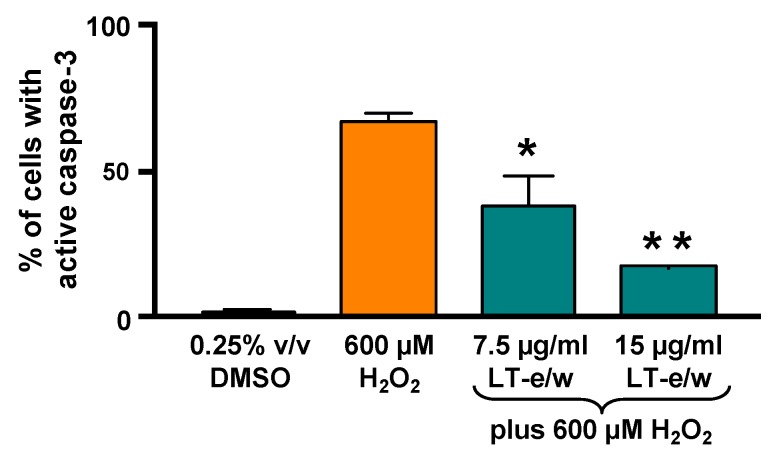
LT-e/w extract mitigated the H_2_O_2_-activated caspase-3 significantly in SH-SY5Y cells in a dose-dependent mode. Cells with active caspase-3 were stained with NucView 488 caspase-3 substrate and analyzed in a live-cell mode by flow cytometer. Each data point signifies the average of three biological replicates with its standard deviation. The asterisk(s) indicate a significant difference between cells treated concomitantly with both 15 µg/mL LT-e/w extract and 600 µM H_2_O_2_, as compared to cells treated with just 600 µM H_2_O_2_; *p* < 0.05 (*) and *p* < 0.01 (**), respectively. The Kaluza flow cytometry software (Beckman Coulter) was employed for acquisition and analysis purposes.

**Figure 8:**
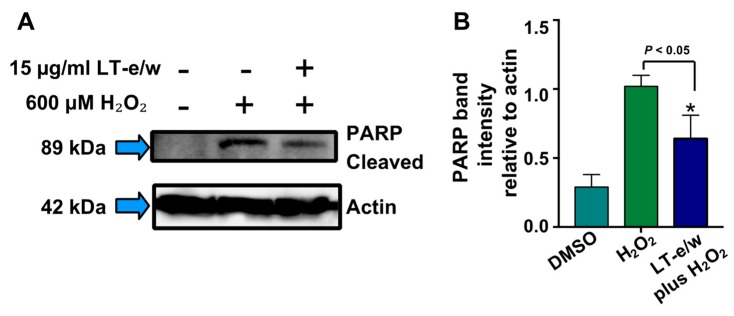
LT-e/w extract attenuates the H_2_O_2_-induced PARP cleavage significantly in SH-SY5Y cells in a dose-dependent modality. Whole-cell lysates, with an equal amount of proteins in each lane, were subjected to western blot analysis for PARP cleavage determinations. (**A**) Representative western blot used for PARP cleavage analysis. (**B**) Each bar in the graph represents the average of densitometry analyses of two biological replicates with its corresponding standard deviation. (**B**) PARP cleaved band intensity values are relative to actin loading control protein. As a reference, lysates from solvent control (DMSO)-treated cells were included. Densitometry analysis was performed using the ImageStudio software suite (LI-COR).

To elucidate whether caspase-3 activation was directly involved during cytoprotection against H_2_O_2_-induced cytotoxicity, SH-SY5Y cells were concomitantly exposed to both LT-e/w extract (7.5 µg/mL and 15 µg/mL) and 600 μM H_2_O_2_. Figure 7 shows significant increases in caspase 3 activity when exposed to 600 µM H_2_O_2_ (*p* < 0.01 compared with DMSO control). Cotreated cells (LT-e/w extract at 7.5 µg/mL and 15 µg/mL and 600 μM H_2_O_2_) showed a significant reduction in caspase 3 activity compared with the 600 μM H_2_O_2_ control (*p* < 0.001 in 15 µg/mL cotreatment compared with 600 μM H_2_O_2_ control). Also, cells co-exposed with LT-e/w extract (15µg/mL) and 600 μM H_2_O_2_ showed a significant reduction in cleaved PARP expression (Figure 8). These observations suggest that LT-e/w extract prevented H_2_O_2_-induced apoptosis in SH-SY5Y cells by reduction of caspase-3 activation.

### 3.7. Analysis of the Cell Cycle Profile by Flow Cytometry

**Figure 9:**
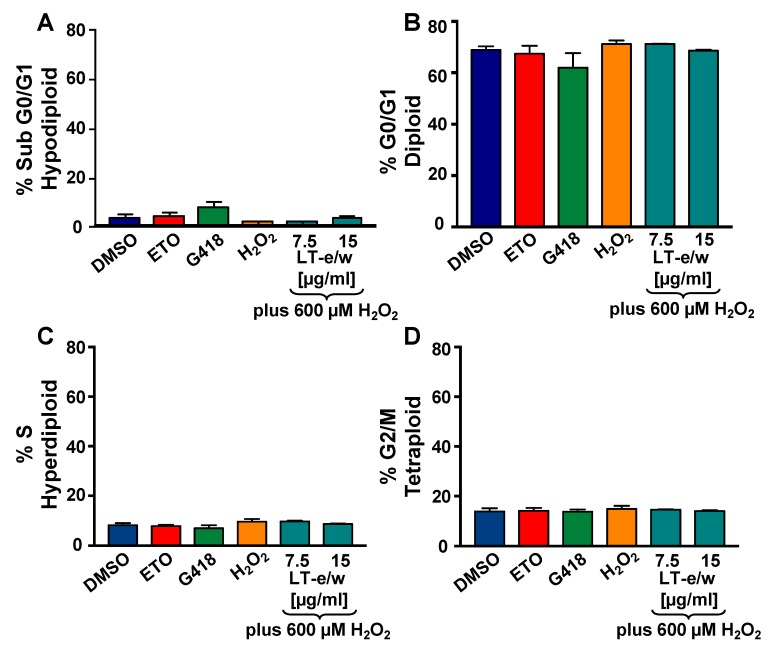
LT-e/w extract did not modify the cell cycle distribution profile or the DNA fragmentation subpopulation in SH-SY5Y cells under oxidative stress. Cells were fixed, permeabilized, and 4′,6-diamidino-2-phenylindole (DAPI)-stained and analyzed via Gallios flow cytometer. (**A**–**D**) The events (cells) frequency are plotted along with the y-axis, whereas the varied treatments are included along with the horizontal x-axis. Controls for this series of experiments included: Cells treated with the solvent control (DMSO), 200 nM etoposide (ETO), 1 mg/mL G418, and 600 µM H_2_O_2_. Each bar represents an average of triplicate and the error bars represent their corresponding standard deviation. For data acquisition and analysis purposes, the Kaluza flow cytometry software was utilized (Beckman Coulter).

To investigate the potential antiproliferative rescue effect of LT-e/w extract based on the cell cycle analysis profile on cells undergoing to oxidative stress, the violet-excited DNA intercalating fluorophore, DAPI (4′,6-diamidino-2-phenylindole), and flow cytometer were used [23,27]. Initially, H_2_O_2_-treated SH-SY5Y cells displayed a similar cell cycle profile as compared with solvent control cells (Figure 9). The percentages for each cell cycle phase subpopulation were similar in both treated and control cells. Also, after simultaneously exposure to both H_2_O_2_ and LT-e/w extract, the cell cycle profile of SH-SY5Y cells remained unaltered (Figure 9). Thus, the cell cycle profile of cells under oxidative stress was unaffected and remained unaltered after co-exposure to both H_2_O_2_ and LT-e/w extract. Moreover, the undetected increase of the sub-G0-G1 subpopulations was interpreted as the absence of DNA fragmentation provoked by oxidative stress alone, as well as in the co-exposure experiments, to both H_2_O_2_ and LT-e/w (Figure 9A) [23,27,28].

## 4. Discussion

In the present study, a creosote bush, *Larrea tridentata* (LT), ethanol-water (e/w; 60:40% *v*/*v*) leaves extract was tested for its cytoprotective effect on neuroblastoma cells undergoing H_2_O_2_-induced oxidative stress in an in vitro model. Thus, to overcome the limitations of using primary cultures of mammalian neurons cells, a well-established model of transformed human neuroblastoma SH-SY5Y cells was utilized [29]. Throughout history, the kingdom Plantae has provided a very important source of natural products adopted by modern drug discovery initiatives, including numerous antioxidant compounds have that has been used as a basis for the development of effective therapeutic drugs [30].

*L. tridentata* is a plant with a remarkable source of natural products extracted from their leaves and resin, including glycosylated flavonoids, sapogenins, essential oils, halogenic alkaloids, and lignans [31]. Recently, we evaluated the antioxidant activity of three leaves extracts of *L. tridentata*, finding that the e/w extract showed the most efficient antioxidant properties with the highest phenolic content [18]. Also, the HPLC-MS study of this e/w extract identified phenolic compounds previously reported in *L. tridentata* [18].

The induction of oxidative stress and apoptosis in SH-SY5Y cells has been previously employed as a successful in vitro model for cytoprotection studies [29,32,33,34,35]. To find the best conditions to evaluate the rescue effect of LT-e/w extract, we first determined the individual cytotoxicity of the LT-e/w extract and the H_2_O_2_ on SH-SY5Y cells (Figure 1A–B). Previously, the LT-e/w extract exhibited low toxicity (96.5%) on non-cancerous Hs27 cell line when tested at 120 µg/mL concentration [18]. In contrast, the LT-e/w extract in this study displayed 57.7% of cytotoxicity at 30 µg/mL concentrations on SH-SY5Y cells (Figure 1A).

Due to the concentration-dependent cytotoxicity observed in LT-e/w-treated cells, we decided to test the cytoprotection of LT-e/w at concentrations lower than 30 µg/mL. Subsequent assays were performed under 12 h incubation and 600 µM H_2_O_2_ conditions, considering the dose-dependent toxicity of the extract and the H_2_O_2_ cytotoxicity at 600 µM (~50%). Previous reports have demonstrated the H_2_O_2_-induced cell apoptosis at concentrations lower than 1 mM and for incubation periods not greater than 24 h [36,37,38]. The cytoprotective effect against H_2_O_2_-induced cell apoptosis in incubation periods of 12 h, and even at shorter periods, has been reported [39,40,41]. Among the three extracts tested, only LT-e/w showed cytoprotective capacity (Figure 2), which was also the one with the most efficient antioxidant properties and the highest phenolic content [18]. It has been reported previously that the total phenol content and the antioxidant activity of extracts are correlated with their cytoprotecting ability [42]. LT-e/w (15 µg/mL) was able to maintain cell viability by about 50% compared to cells treated only with hydrogen peroxide (Figure 2B). Extracts of Chinese medicinal plants (*Astragalus membranaceus*, *Gynostemma pentaphyllum*, and *Lycium barbarum*) have also presented this cytoprotective effect at similar range concentrations of 15 µg/mL ± 5 [42].

The chemoprotective properties exerted by bioactive components existing in vegetables, spices, and fruits, displaying antioxidant and anti-cancer effects, are attributable to the additive and/or synergistic effect of their phytochemical compound-complex mixture [43]. For example, extracts obtained from whole apples (*Malus pumila*; “Red Delicious”) exhibited higher antioxidant and anti-cancer proliferative activities when compared with extracts from the same apples without skin [44]. Moreover, the total polyphenols extracted from cranberries (*Vaccinium macrocarpon*; Ait) displayed better antiproliferative effect on cancer cells as compared with total cranberry extracts, as well as its single components (e.g., anthocyanins, proanthocyanidins, isoflavones, glycosides) [45]. In this study, four fractions isolated from LT-e/w extract were tested for their capacity to improve the viability of cells under oxidative stress. However, none of the four fractions displayed a distinguished rescue activity in the viability of the SH-SY5Y cells. Thus, findings suggest that an additive and/or synergistic interaction of the bioactive components into LT-e/w extract are responsible for the enhanced cytoprotective activity.

Mitochondria are the most important source of radical oxygen species (ROS) production within mammalian cells and, therefore, play a crucial role in generating oxidative stress [46]. In addition, H_2_O_2_ is a dominant mitochondrial endogenous ROS which has been associated with many human chronic neurodegenerative disorders [47]. Also, H_2_O_2_ has been commonly used as a standard reagent to provoke exogenous oxidative stress [48]. Furthermore, it has been observed cells treated with exogenous H_2_O_2_ experienced mitochondrial membrane potential (*ΔΨm*) depolarization, leading to an increase in permeability changes and Cyt-c release [49]. Moreover, the mitochondrial depolarization is an early biochemical event of the apoptotic program occurring before of caspase-3/7 activation [50]. Numerous reports have routinely used the H_2_O_2_ as a positive reagent to inflict successfully *ΔΨm* depolarization [23,27,51,52,53,54]. Additionally, a previous report indicated that a fox grape (*Vitis labrusca*) seed extract was able to prevent cellular morphological alterations, ameliorate mortality, and decrease the levels of apoptosis and necrosis in neuroblastoma SH-SY5Y cells undergoing H_2_O_2_-induced oxidative stress [55]. It was also revealed that a rosemary (*Rosmarinus officinalis*) extract was capable of reducing the mitochondrial depolarization-mediated apoptotic cell death in neuroblastoma SH-SY5Y cells upon H_2_O_2_-induced aggression [47]. In another study, *Artemisia amygdalina* (wormwood) extract protected neurons against mitochondrial membrane potential loss and attenuated reactive oxygen species production [47,56]. Furthermore, an extract of cyanobacteria (*Aphanizomenon flos-aquae*) showed a protective role against neurodegeneration on neuroblastoma LAN5 cell line model [57]. In this report, the capacity of LT-e/w extract to attenuate the H_2_O_2_-provoked mitochondria depolarization in SH-SY5Y cells was tested and monitored using a JC-1 polychromatic reagent and flow cytometer. Findings indicated that LT-e/w extract was able to diminish the H_2_O_2_-elicited *ΔΨm* depolarization on neuroblastoma SH-SY5Y cells in a concentration-dependent manner and significantly reduce the ROS accumulation in SH-SY5Y cells under H_2_O_2_-induced oxidative stress (Figure 4 and Figure 5).

In our study, annexin V-FITC/PI staining assay and flow cytometer demonstrated that the concentration of H_2_O_2_ in used cytoprotection assays is preferential in inducing apoptosis (Figure 6A,C). Furthermore, LT e/w (15 µg/mL) was able to reduce the H_2_O_2_-induced apoptosis by 53% (Figure 6D). Next, we explored the potential effect of LT-e/w extract in decreasing the caspase-3/7 activation triggered by oxidative stress. The caspase family of cysteine proteases is implicated in the executory phases of apoptotic programmed cell death [58]. Activation of caspase-3 is the point of common convergence for both extrinsic and intrinsic apoptotic pathways that were initiated upstream [59]. Once activated, both caspase-3 and -7 are implicated in the execution of the apoptotic cell death. Several preclinical studies reported the ability of various plant extracts to inhibit the caspase apoptotic cascade through direct interaction with caspase proteins [60]. To investigate whether the LT-e/w extract was capable of decreasing the levels of H_2_O_2_-provoked caspase-3/7 activation, SH-SY5Y cells and a live-cell permeable NucView 488 caspase-3 substrate (Biotium), designed for detecting both caspase-3 and -7 activations, was utilized [51,52,54]. Furthermore, once that caspase-3/7 are activated, they cleavage the NucView 488 substrate, releasing a non-fluorescent dye, which upon binding to the DNA, emits a green fluorescent signal. Consequently, cells experiencing the biochemical event of caspase-3/7 activation are easily detectable via flow [23]. Therefore, findings exhibited that LT-e/w extract was able to significantly decrease the levels of caspase-3/7 activation in a dose-dependent manner in SH-SY5Y cells experiencing oxidative stress-induced apoptosis, thus mitigating the oxidative stress-induced cell death (Figure 7).

Once that caspase-3 and caspase-7 are activated, their downstream substrate is poly (ADP-ribose) polymerase (PARP), which is proteolytic cleavage releasing its large 89 kDa fragment and easily detectable via western blot analysis [51]. Both caspases and PARP are all essential key effectors in the execution facet of apoptosis-mediated cell death [61]. Moreover, a biochemical hallmark for the identification of cells undergoing apoptosis is the proteolytic cleavage of PARP [62]. PARP is a nuclear DNA enzyme that is in charge of repairing and maintaining genome integrity [63]. Furthermore, the caspases-3/7-mediated PARP cleavage has been associated with multiple neurological illnesses, including Alzheimer’s and Parkinson’s disease, as well as multiple sclerosis, traumatic brain injury, and brain tumors [64]. Also, it is well-known that H_2_O_2_-induced oxidative stress increases the PARP cleavage levels. Thus, in this study, we tested whether LT-e/w extract was able to reduce the levels of PARP cleavage in neuroblastoma SH-SY5Y cells undergoing oxidative stress (Figure 8). These findings indicated that LT-e/w extract was able to significantly reduce the H_2_O_2_-induced PARP cleavage in a dose-dependent mode.

To further examine the LT-e/w extract effect as a regulator of cell proliferation, we next selected to analyze the cell cycle profile of the SH-SY5Y cell under oxidative stress. For this purpose, the total cellular DNA content strategy and a nucleic acid intercalator reagent (DAPI) were used to examine each cell cycle facets via flow cytometer. The Gallios flow cytometer was equipped with a solid-state 405 nm wavelength violet laser proficient at exciting the DAPI dye [23,27]. When DAPI is intercalated into the DNA, its fluorescence signal emission maximum is ~461 nm, and when DAPI is intercalated with RNA, its emission maximum is ~500 nm [23]. The Gallios flow cytometer used in this series of experiments was able to capture only the fluorescence signal emitted by DNA-DAPI complexes (~461 nm), discarding the signal emitted by RNA-DAPI complexes (~500 nm), when using the FL9 detector. Therefore, using this approach, the elimination of RNA using enzymatic digestion (RNase) is unnecessary [23,27]. Also, the time involved in the permeabilization and staining steps used for sample preparation takes 3 min after single-reagent-addition to the cell suspension (nuclear isolation medium (NIM)-DAPI solution; Beckman Coulter). Thus, findings suggest that the cell cycle profile of SH-SY5Y cells under oxidative stress was similar to the solvent controls and remained unaffected after concomitant exposure to both H_2_O_2_ and LT-e/w extract (Figure 9). Additionally, the DNA fragmentation was also undetected in the experimental samples, as evidenced by the lack of increase of the sub-G0/G1 subpopulations (Figure 9A) [23,27,28]. The absence of alterations of the cell cycle distribution profile was likely due to the short incubation periods used for the cell cycle analyses.

## 5. Conclusions

**Figure 10:**
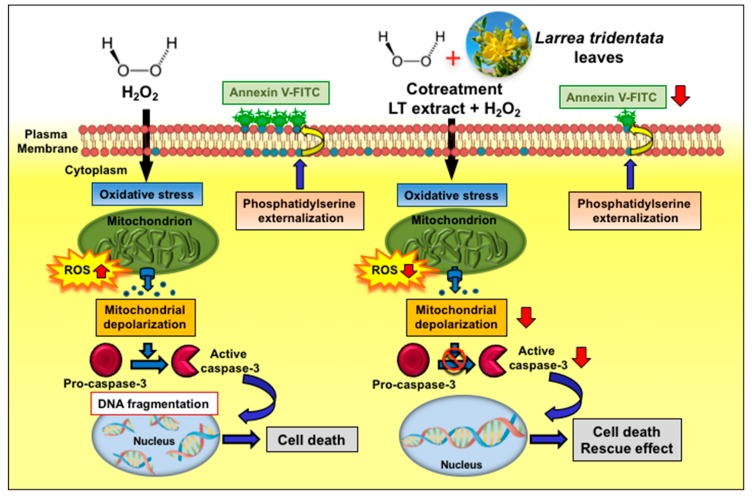
Scheme summarizing the cytoprotecting mechanism of action of *Larrea tridentata* e/w extract against oxidative stress. LT-e/w extract decreases the levels of H_2_O_2_-provoked ROS accumulation, mitochondrial depolarization, phosphatidylserine externalization, caspase-3/7 activation, and PARP cleavage significantly.

In this study, our results revealed that LT-e/w leaves extract possess an antioxidant rescue effect, mitigating the aggressive cytotoxicity provoked by H_2_O_2_-induced oxidative stress on human neuroblastoma SH-SY5Y cells, as evidenced by improving their cell viability. Additionally, we investigated the potential mechanism implicated in this rescue activity, finding that LT-e/w extract reduced the levels of H_2_O_2_-triggered ROS accumulation, mitochondrial depolarization, phosphatidylserine externalization, caspase-3/7 activation, and PARP cleavage significantly, which are hallmarks of apoptosis (Figure 10). In contrast, alterations of all the facets of the cell cycle profile were undetected in both experimental and control cells. Hence, these findings demonstrate that the LT-e/w extract exerted a favorable cytoprotective activity against oxidative stress in vitro, acting as an anti-apoptotic reagent. Furthermore, LT-e/w extract could be used as a potential natural antioxidant that may be advantageous, contributing to the oxidative stress-elicited illnesses chemotherapeutic initiatives.

## Supplementary Materials

The following are available online at https://www.mdpi.com/2076-3921/8/10/427/s1, Figure S1. Cytotoxicity of H_2_O_2_ on SH-SY5Y cells. Figure S2. LT extracts prepare with three different solvents were tested for 12 h on SH-SY5Y cells under 300 µM H_2_O_2_-induced oxidative stress. Figure S3. LT extracts prepare with three different solvents were tested for 18 h on SH-SY5Y cells under 300 µM H_2_O_2_-induced oxidative stress. Figure S4. LT extracts prepare with three different solvents were tested for 24 h on SH-SY5Y cells under 300 µM H_2_O_2_-induced oxidative stress. Figure S5. LT extracts prepare with three different solvents were tested for 12 h on SH-SY5Y cells under 150 µM H_2_O_2_-induced oxidative stress. Figure S6. LT extracts prepare with three different solvents were tested for 18 h on SH-SY5Y cells under 150 µM H_2_O_2_-induced oxidative stress.
