# Supplementary material for: Larrea tridentata Extract Mitigates Oxidative Stress-Induced Cytotoxicity in Human Neuroblastoma SH-SY5Y Cells
Source: Antioxidants (Basel). 2019 Sep 25;8(10):427. doi: 10.3390/antiox8100427 (PMC6827101; doi:10.3390/antiox8100427)
Supplement: Supplementary file 1 [file antioxidants-08-00427-s001.pdf]

antioxidants

MDPI

Supplementary Material

Larrea tridentata extract mitigates oxidative stress-induced cytotoxicity in human neuronal SH-SY5Y cells

Karla Morán-Santibañez[1], Abimael H. Vasquez[1], Armando Varela-Ramirez[2], Veronica Henderson[3], Janae Sweeney[3], Valerie Odero-Marah[3], Karine Fenelon[1,4,] *, Rachid Skouta[1,4,] *.

[1] Department of Chemistry and Biochemistry, Border Biomedical Research Center, The University of Texas at El Paso, El Paso, TX 79968, USA; ksmoransant@utep.edu (K.M.-S.) ORCID ID https://orcid.org/0000-0002-3886-8599; ahvasquez@miners.utep.edu (A.H.V.)
[2] Border Biomedical Research Center (BBRC), Department of Biological Sciences, the University of Texas at El Paso, El Paso, TX 79968, USA; avarela2@utep.edu (A.V-R) ORCID ID https://orcid.org/0000-0002-2071-4874.
[3] Department of Biological Sciences, Center for Cancer Research and Therapeutic Development, Clark Atlanta University, Atlanta GA 30314; vhenderson@cau.edu (V.H.); janae.sweeney@students.cau.edu (J.S.); voderomarah@cau.edu (V. O.-M.)
[4] Department of Biology, University of Massachusetts, Amherst, MA 01003-9297, USA.

*****Correspondence: rskouta@umass.edu (R.S.); kfenelon@umass.edu (K.F.); Tel.: +1-915-747-5318 (R.S.); +1-915-747-8757 (K.F.)

TABLE OF CONTENT

Page 2. Figure S1. Cytotoxicity of H_2O_2 on SH-SY5Y cells.

Page 3. Figure S2. LT extracts prepare with three different solvents were tested for 12 h on SH-SY5Y cells under 300 µM H_2O_2-induced oxidative stress.

Page 4. Figure S3. LT extracts prepare with three different solvents were tested for 18 h on SH-SY5Y cells under 300 µM H_2O_2-induced oxidative stress.

Page 5. Figure S4. LT extracts prepare with three different solvents were tested for 24 h on SH-SY5Y cells under 300 µM H_2O_2-induced oxidative stress.

Page 6. Figure S5. LT extracts prepare with three different solvents were tested for 12 h on SH-SY5Y cells under 150 µM H_2O_2-induced oxidative stress.

Page 7. Figure S6. LT extracts prepare with three different solvents were tested for 18 h on SH-SY5Y cells under 150 µM H_2O_2-induced oxidative stress.

CONTENT

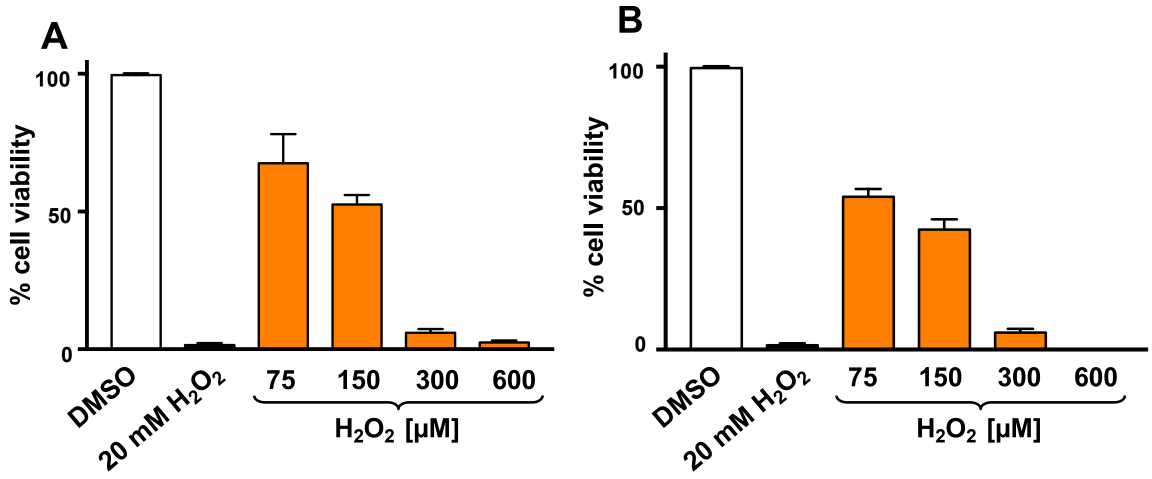

Figure S1. Cytotoxicity of H_2O_2 on SH-SY5Y cells. The percentage of viable cells was measured by using the differential nuclear staining (DNS) assay and a bioimager system. Cells were exposed for **A**) **18 h** and **B**) **24 h** to an H_2O_2 concentration gradient (75 to 600 μM) and their cytotoxicity was determined. DMSO 0.25% v/v was included as solvent control, and as a positive control for cytotoxicity, 20 mM of H_2O_2-treated cells were also included. Each bar indicates the average of three biological replicates with its corresponding standard deviation.

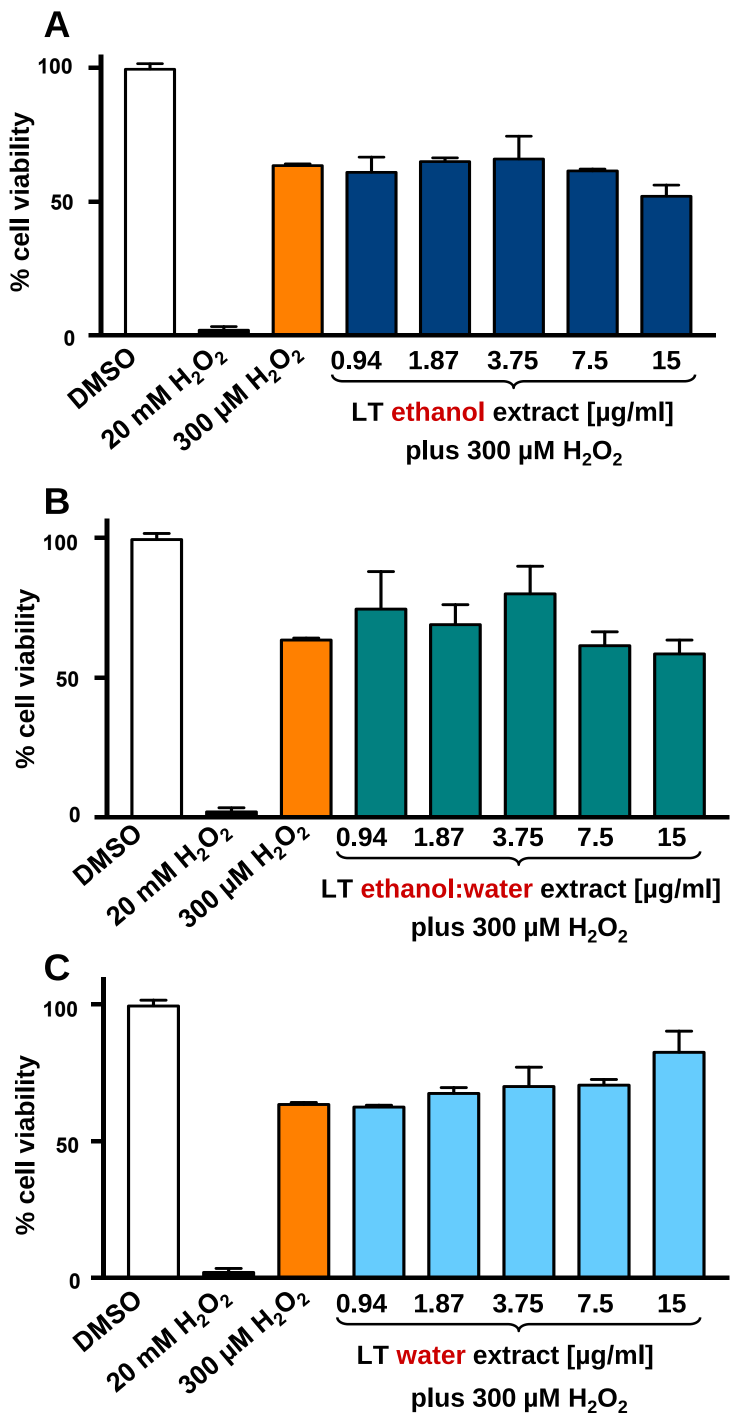

Figure S2. LT extracts prepare with three different solvents were tested for 12 h on SH-SY5Y cells under 300 µM H_2O_2-induced oxidative stress. To quantify the cell viability, the differential nuclear staining (DNS) assay and a bioimager system were used. Cells were concurrently exposed for **12 h** to both a single **300 µM** H_2O_2 concentration and a concentration gradient (0.94 to 15 µg/ml) of the LT extracts in different solvents: **A**) ethanol, **B**) ethanol:water (e/w) mixture and **C**) water. Controls included cells treated with just DMSO (0.25% v/v) or 20 mM of H_2O_2 as positive for cytotoxicity. The asterisk (*) is indicating a significant difference between cells treated with both LT-e/w extract and H_2O_2 (300 µM), as compared with cells treated with just 300 µM H_2O_2 control ($P < 0.05$). Each bar is showing the average of three biological replicates with its corresponding standard deviation.

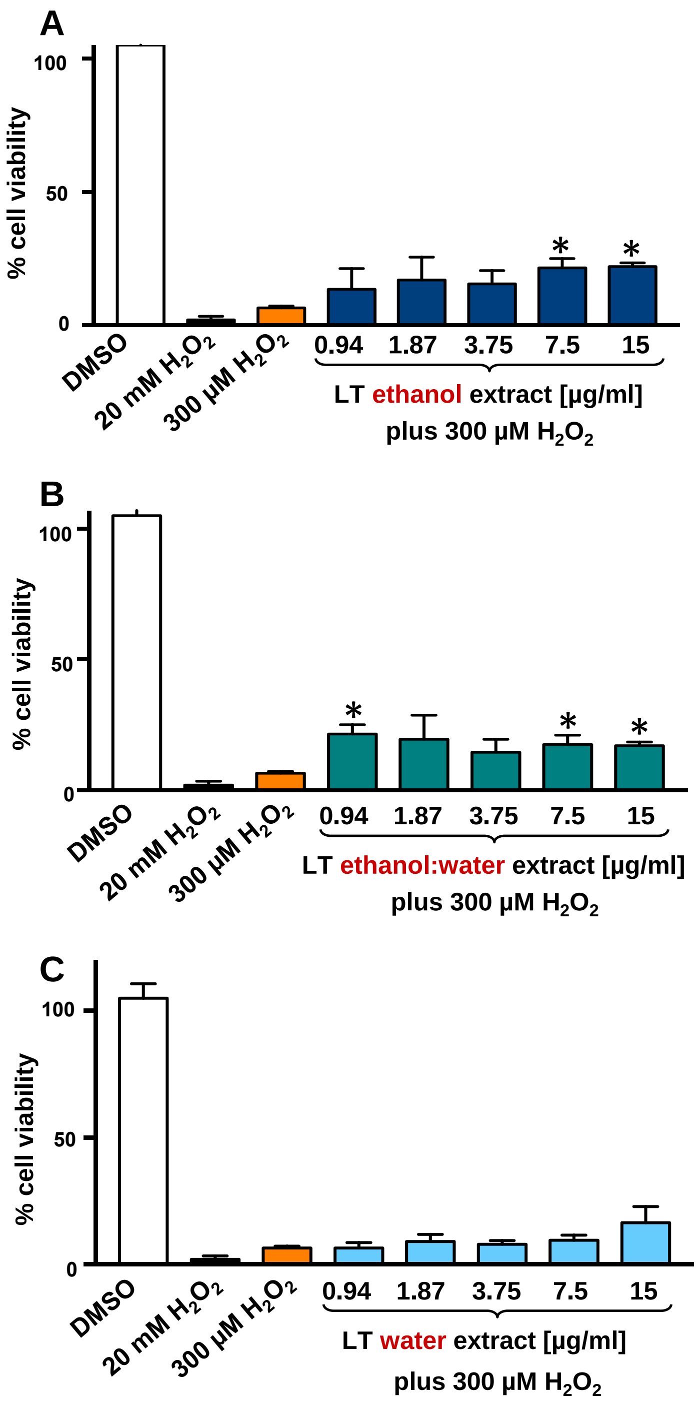

Figure S3. LT extracts prepare with three different solvents were tested for 18 h on SH-SY5Y cells under 300 µM H_2O_2-induced oxidative stress. To quantify the cell viability, the differential nuclear staining (DNS) assay and a bioimager system were used. Cells were exposed for **18 h** to both a single **300 µM** H_2O_2 concentration and a concentration gradient (0.94 to 15 µg/ml) of the LT extracts in different solvents: **A**) ethanol, **B**) ethanol:water (e/w) mixture and **C**) water. Controls included cells treated with just DMSO (0.25% v/v) or 20 mM of H_2O_2 as positive for cytotoxicity. The asterisk (*) is indicating a significant difference between cells simultaneously treated with both LT-e/w extract and H_2O_2 (300 µM), as compared with cells treated with just 300 µM H_2O_2 control ($P < 0.05$). Each bar is showing the average of three biological replicates with its corresponding standard deviation.

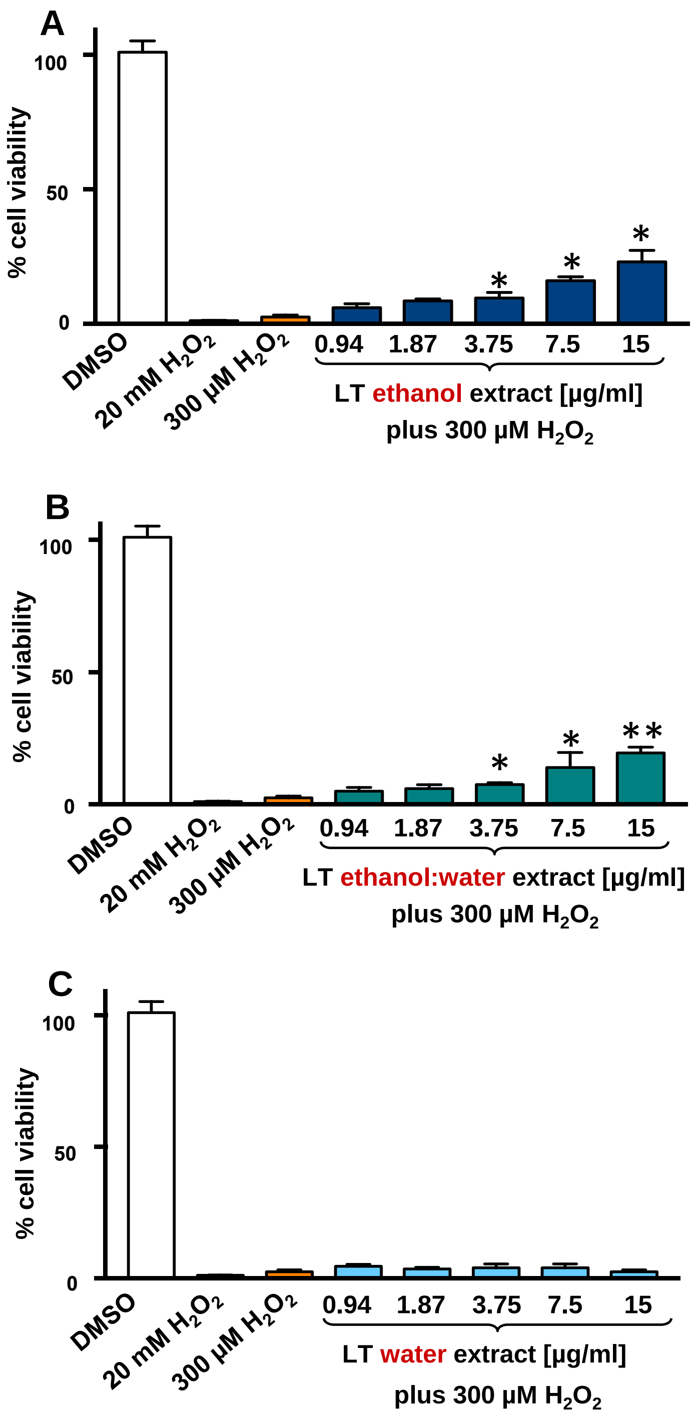

Figure S4. LT extracts prepare with three different solvents were tested for 24 h on SH-SY5Y cells under 300 µM H_2O_2-induced oxidative stress. To quantify the cell viability, the differential nuclear staining (DNS) assay and a bioimager system were used. Cells were concurrently exposed for **24 h** to both a single **300 µM** H_2O_2 concentration and a concentration gradient (0.94 to 15 µg/ml) of the LT extracts in different solvents: **A**) ethanol, **B**) ethanol:water (e/w) mixture and **C**) water. Controls included cells treated with just DMSO (0.25% v/v) or 20 mM of H_2O_2 as positive for cytotoxicity. The asterisk(s) is indicating a significant difference between cells treated with both LT-e/w extract and H_2O_2 (300 µM), as compared with cells treated with just 300 µM H_2O_2 control; $P < 0.05$ (*) and $P < 0.01$ (**), respectively. Each bar is showing the average of three biological replicates with its corresponding standard deviation.

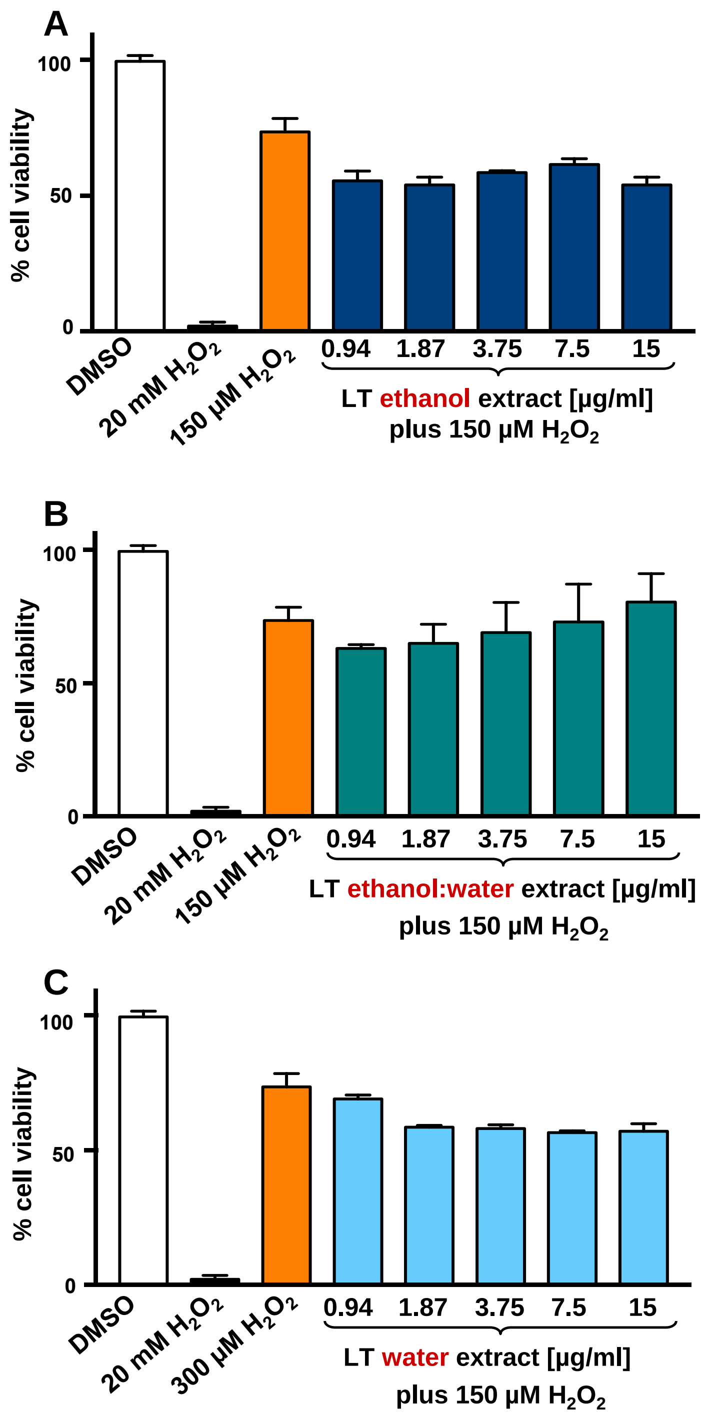

Figure S5. LT extracts prepare with three different solvents were tested for 12 h on SH-SY5Y cells under 150 µM H_2O_2-induced oxidative stress. To quantify the cell viability, the differential nuclear staining (DNS) assay and a bioimager system were used. Cells were simultaneously exposed for **12 h** to both a single **150 µM** H_2O_2 concentration and a concentration gradient (0.94 to 15 µg/ml) of the LT extracts in different solvents: **A**) ethanol, **B**) ethanol:water (e/w) mixture and **C**) water. Controls included cells treated with just DMSO (0.25% v/v) or 20 mM of H_2O_2 as positive for cytotoxicity. Each bar is showing the average of three biological replicates with its corresponding standard deviation.

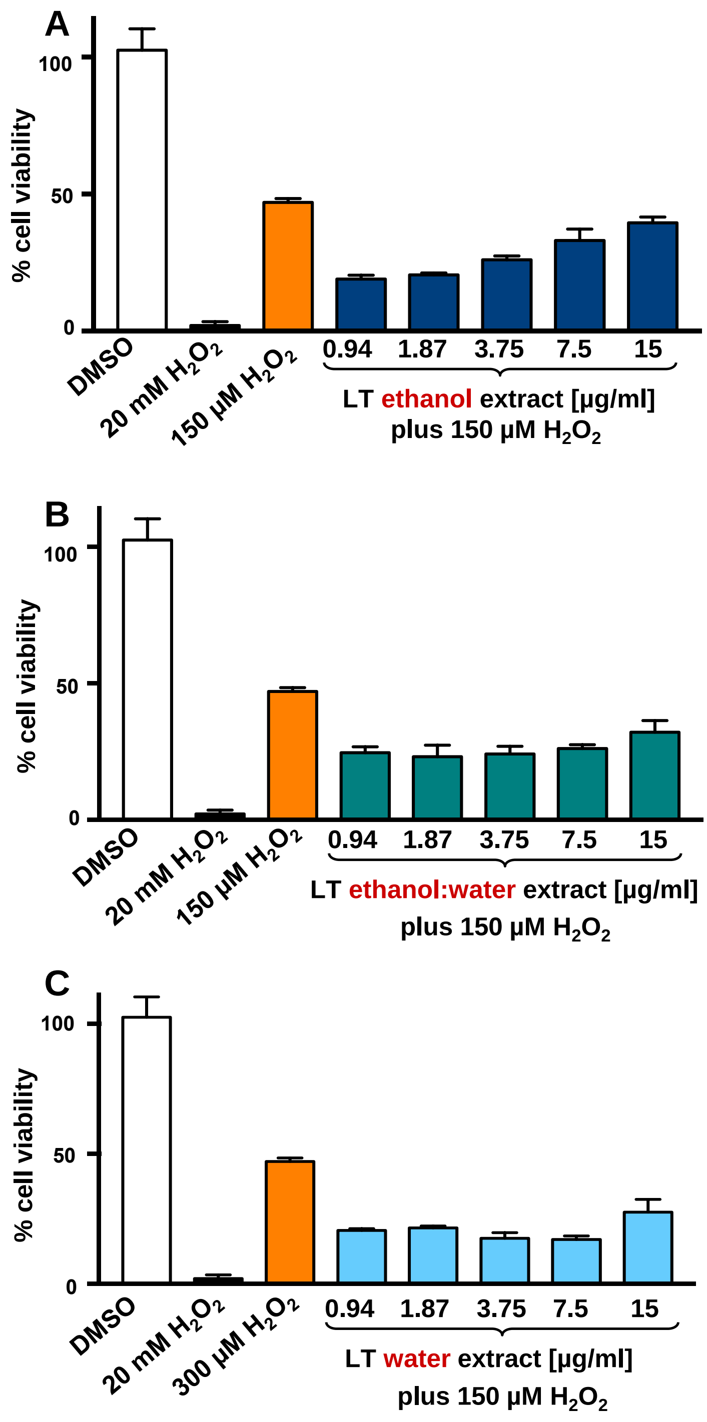

Figure S6. LT extracts prepare with three different solvents were tested for 18 h on SH-SY5Y cells under 150 μM H_2O_2-induced oxidative stress. To quantify the cell viability, the differential nuclear staining (DNS) assay and a bioimager system were used. Cells were concomitantly exposed for **18 h** to both a single **150 μM** H_2O_2 concentration and a concentration gradient (0.94 to 15 μg/ml) of the LT extracts in different solvents: **A**) ethanol, **B**) ethanol:water (e/w) mixture and **C**) water. Controls included cells treated with just DMSO (0.25% v/v) or 20 mM of H_2O_2 as positive for cytotoxicity. Each bar is showing the average of three biological replicates with its corresponding standard deviation.
